# Characteristics and perioperative complications of hip fracture in the elderly with acute ischemic stroke: a cross-sectional study

**DOI:** 10.1186/s12891-022-05585-2

**Published:** 2022-07-05

**Authors:** Yaqian Zhang, Mingming Fu, Junfei Guo, Yuqi Zhao, Zhiqian Wang, Zhiyong Hou

**Affiliations:** 1 Department of Geriatric Orthopedics, The Third Hospital of Hebei Medical University, Shijiazhuang, Hebei 050051 P.R. China; 2 Department of Orthopaedic Surgery, The Third Hospital of Hebei Medical University, Shijiazhuang, Hebei 050051 P.R. China; 3 NHC Key Laboratory of Intelligent Orthopeadic Equipment (The Third Hospital of Hebei Medical University), Shijiazhuang, P.R. China

**Keywords:** Hip fracture, Ischemic stroke, Characteristics, Complications

## Abstract

**Background:**

Patients with acute ischemic stroke (AIS) after hip fracture in the elderly have worse prognosis. We aimed to describe the characteristics and complications of hip fracture with AIS in the elderly.

**Methods:**

This cross-sectional study selected patients with hip fracture (age ≥65 years) from January 2018 to September 2020. The collected data included age, sex, fracture types, comorbidities. In above screened patients, we further collected cerebral infarction related information of AIS patients. The least absolute shrinkage and selection operator (LASSO) logistic regression was performed to identify the strongest predictors of AIS after hip fracture. Multivariate logistic regression analysis was conducted to find independent risk factors for AIS after hip fracture.

**Results:**

Sixty patients (mean age 79.7 years;female 56.7%) occurred AIS after hip fracture in 1577 cases. The most common infarction type was partial anterior circulation infarction (PACI) (70.0%). The majority of these infarction lesions were single (76.7%) and most infarction lesions(65.0%) were located in the left side. 81.7% of AIS patients had mild (Health stroke scale NIHSS <4) AIS. Older patients with AIS after hip fracture were more frequently complicated by hypertension(73.3%), prior stroke (46.7%), diabetes(35.0%) and were more likely to have hypoproteinemia(68.3%), electrolyte disorders ( 66.7%), anemia (65.0%), deep vein thrombosis (51.6%), pneumonia (46.6%),cardiac complications (45.0%). Combined with hypertension (OR 2.827, 95%CI 1.557-5.131) and male sex(OR 1.865, 95%CI 1.095-3.177) were associated with the increased risk of AIS after hip fracture.

**Conclusions:**

Older patients combined with hypertension are more likely to have AIS after hip fracture. For these patients, early preventions should be administered. AIS patients after hip fracture are prone to have multiple complications under traumatic stress, and we should enhance the management of these patients to reduce the stress and avoid occurrence of complications.

## Introduction

As the accelerated rate of aging society, the number of older patients with hip fractures continually increase, which has become a global medical and health problem [1, 2]. There are approximately 1.5 million femoral fractures worldwide each year, and this number is predicted to rise to 6.3 million by 2050 [3]. Hip fractures of the older adults, which are often referred to "the last fracture of life ", severely affect limb function and quality of life, and greatly increase social and family burdens [4]. A report showed that older patients have a high mortality of 8.4-36% within one year after the fracture [5].

Previous studies have found that complications were a substantial contributor to high mortality in older patients with hip fracture [6, 7]. AIS is one of the catastrophic complications and associated with increased mortality in the hip fracture patients [8]. Fracture patients with perioperative AIS have worse functional recovery than those with non-AIS and require more care in the first year [9]. Currently, the existing studies focus on the occurrence of stroke in the perioperative period and 1 year after the surgeries of hip fracture [10–12], while there are few studies on AIS after fracture. AIS after hip fracture makes patients unable to perform surgery in a short time and results in poor outcomes including impaired self-care, decreased quality of life and increased mortality [13]. Due to blood loss, pain and other factors, elderly patients with hip fracture are prone to traumatic stress reactions [14], which increase the risk of complications. We should pay more attention to these patients. However, there are few studies on the complications of AIS patients after hip fracture.

LASSO regression is a popular high-dimensional prediction regression method [15]. In this study, we retrospectively described characteristics and complications of hospitalized older adults with hip fractures who sustained AIS. We also estimated the proportion of AIS after hip fracture and constructed LASSO logistic regression model and multivariate logistic regression to evaluate risk factors for AIS after hip fracture in elderly patients. These results will help people to understand characteristics of older hip fracture adults with AIS and provide treatment recommendations for these specific populations.

## Materials and methods

### Patients and groups

In this retrospective study, we collected data in a single Level I trauma center in a city in China from January 2018 to September 2020. The study protocol was approved by the institutional review board of the third Hospital of Hebei Medical University, and an exemption from the informed consent was obtained. Inclusion criteria were hip fracture patients aged 65 years or older and complete data available in medical records. The exclusion criteria were as follows: (1) had multiple fracture; (2) had pathological fracture, (3) had periprosthetic fracture (4) incomplete medical information. Diagnosis of AIS should be based on consciousness, physical performance, head CT, and MRI performance. All AIS patients fulfilled the World Health Organization criteria for AIS [16]. Patients were retrospectively divided into 2 groups: those who developed AIS (AIS group) and those without AIS (No-AIS group).

## Data collection

The collected data of screened patients included sex, age, and fracture types, comorbidities in both groups. In AIS group, we further collected cerebral infarction related information. AIS was further classified into four subtypes as follows: total anterior circulation infarction (TACI), PACI, posterior circulation infarction (POCI), and lacunar infarction (LACI). Infarction lesions were classified into single infarction lesion and multiple infarction lesions. The sides of cerebral infarction included left side, right side or both sides. Severity of stroke was determined with the National Institutes of NIHSS and categorized as mild (NIHSS<4), moderate (NIHSS 4–15), and severe (NIHSS >15) stroke.

### Statistical analysis

Continuous parametric data was expressed as the mean ± standard deviation and analyzed by student’s t test or analysis of variance. Categorical variables were expressed as numbers and percentages and were compared using chi-squared test or Fisher’s exact test. The LASSO logistic regression model was used to identify the strongest predictors from plenty of potential risk factors for targeted outcome with relatively small quantity. The selected factors were then included in multivariate logistic regression analysis to explore independent risk factors of AIS after hip fracture. IBM SPSS software 26.0 (IBM Corp, Armonk, NY) and R software version 4.1.2 and the “glmnet” package (R Foundation for Statistical Computing, Vienna, Austria) were used to carry out all statistical analyses.

## Results

### Characteristics of AIS

Table 1 showed that the types of AIS in elderly hip fracture patients with AIS were PACI (42, 70.0%), POCI (9, 15.0%), LACI (8, 13.3%),TACI (1, 1.7%).There were 46(76.7%) cases of solitary lesion and 14(23.3%) cases of multiple cerebral infarction and most of them were located on the left side(65%). In addition, in these cases, there were 49(81.7%), 9(15.0%) and 2(3.3%) patients with mild (NIHSS<4), moderate (NIHSS 4–15), and severe (NIHSS >15) stroke, respectively.

**Table 1:** The characteristics of AIS

	Number (n)	Percentage(%)
**Type of cerebral infarction**		
PACI	42	70.0
POCI	9	15.0
LACI	8	13.3
TACI	1	1.7
**Distribution of cerebral infarction lesions**		
Single infarction lesion	46	76.7
Multiple infarction lesion	14	23.3
**Side of cerebral infarction**		
Left side	39	65.0
Right side	18	30.0
Both sides	3	5.0
**NIHSS score**		
<4	49	81.7
4-15	9	15.0
>15	2	3.3

Values are presented as the number (n) and percentage(%)

### The demographic characteristics of all patients

**Fig. 1:**
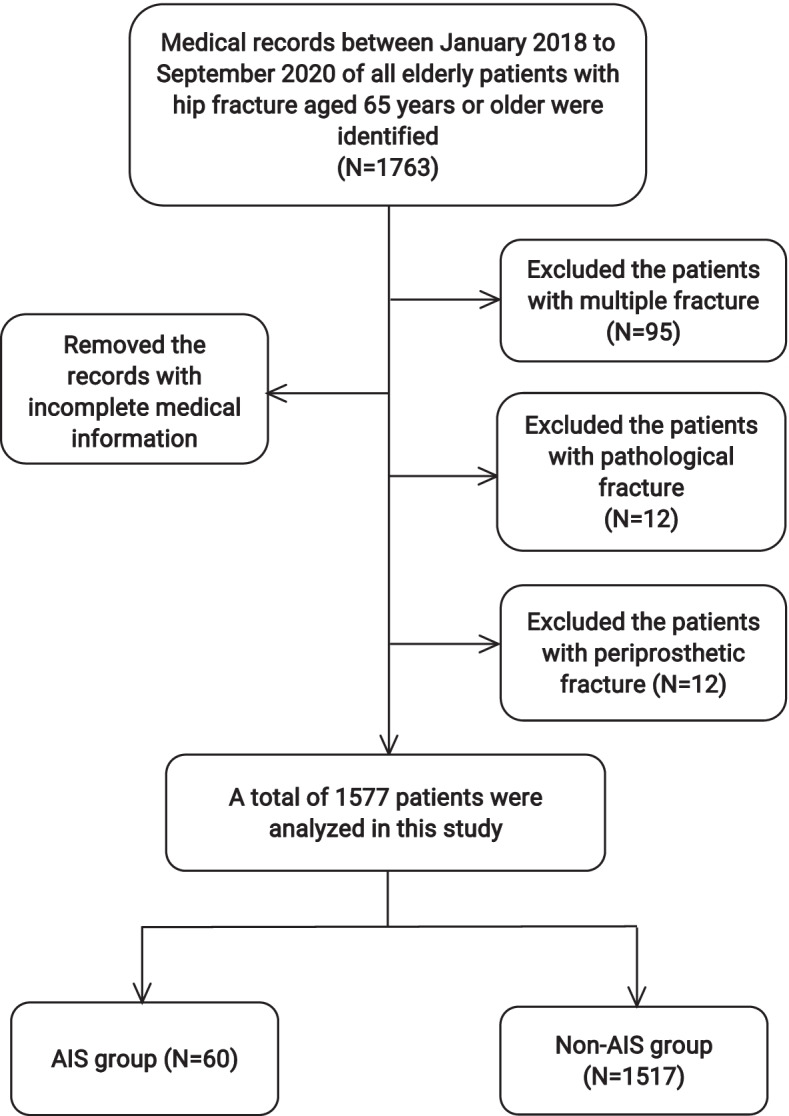
The flow diagram of this study

As shown in Fig. 1, 1763 patients were selected in our study, of these, 186 were excluded from the analysis. Ultimately, a total of 1577 eligible participants met inclusion and were included in our analysis. There were 483 males and 1094 females in this study. The average age was 79.7 years old (ranging from 65 to 104 years old). The proportion of AIS after hip fracture was 3.8%. The proportion of male in the AIS group was higher than that of the non-AIS group (43.3% vs 30.1%, *p*=0.029). AIS patients had more comorbidities( number≥3, *p*=0.039), including hypertension (*p*=0.000) and diabetes (*p*=0.035). In addition, among these patients, the most common comorbidity was hypertension, which was presented in 50.5 % of the patients. Other common comorbidities included prior stroke (41.3%), coronary heart disease (29.4%), diabetes (23.7%). This information can be seen in Table 2.

**Table 2:** The Comparison of the AIS group and No-AIS group

	Overall ((*n*=1577)	AIS (*n*=60)	No-AIS (*n*=1517)	*p* Value
**Age (year)**				
Mean years	79.7±7.3	79.8±6.9	79.7±7.3	0.217
**Gender n (%)**				
Male	483(30.6)	26(43.3)	457 (30.1)	**0.029**
Female	1094(69.4)	34(56.7)	1060(69.9)	
**Fracture type n (%)**				
Neck of femur	758(48.1)	27(45.0)	731(48.2)	0.628
Intertrochanteric	819(51.9)	33(55.0)	786(51.8)	
**Comorbidities n (%)**				
Hypertension	796 (50.5)	44(73.3)	752(49.6)	**0.000**
Prior stroke	652(41.3)	28(46.7)	624(41.1)	0.393
Coronary heart disease	464(29.4)	14(23.3)	450(29.7)	0.291
Diabetes	373(23.7)	21(35.0)	352(23.2)	**0.035**
Arrhythmia	94(6.0)	5(8.3)	89(5.9)	0.429
Pulmonary disease	48(3.0)	4(6.7)	44(2.9)	0.106
Tumor	74(4.7)	4(6.7)	70(4.6)	0.461
Comorbidities≥3	354(22.4)	20(33.3)	334(22.0)	**0.039**

Values are presented as the number (n) and percentage(%) *p*< 0.05, statistical significance

### Risk factors of AIS after hip fracture

**Fig. 2:**
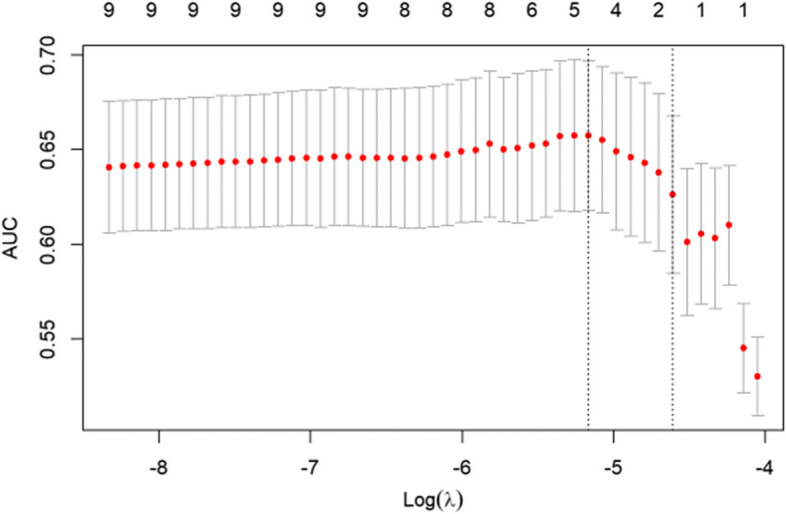
The cross-validation results of LASSO-Logistic regression

**Fig. 3:**
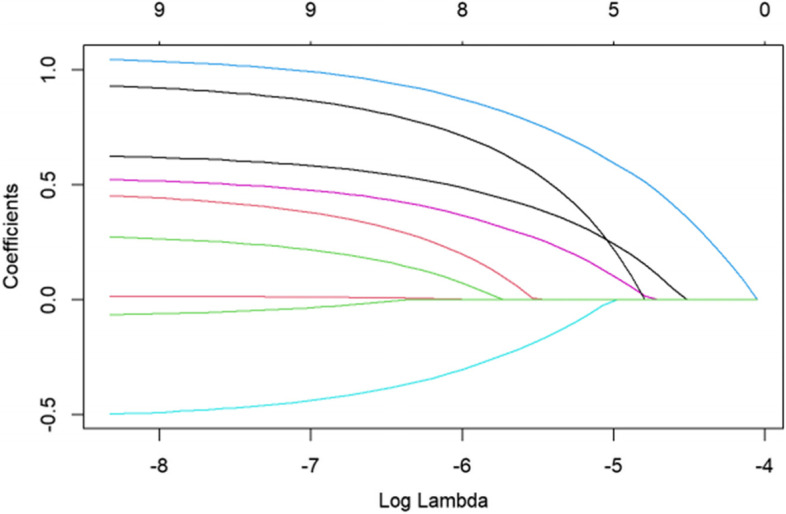
LASSO coefficient profiles of the variables

The LASSO logistic model was subjected to a generalised cross validation. With the change in the log (λ) value of the harmonic parameter, the area under the curve (AUC) value of the ordinate model also changed. The number of corresponding variables screened out by the model is listed in Fig. 2. We used a LASSO logistic regression model to build a risk factor classifier (Fig. 3). After the LASSO logistic analysis, five risk factors were selected, including hypertension, male sex, coronary heart disease, diabetes, pulmonary disease (Table 3). Coefficients for these factors were shown as follows: 0.304 for male, 0.658 for hypertension, -0.061 for coronary heart disease, 0.167 for diabetes and 0.357 for pulmonary disease. Table 4 shows the results of the multivariable analyses. In the whole sample, combined with hypertension (OR 2.827, 95%CI 1.557-5.131) and male sex (OR 1.865, 95%CI 1.095-3.177) were associated with the increased risk of AIS after hip fracture.

**Table 3:** Risk factors selected by LASSO-logistic regression model

Variables	Risk factors	Coefficient
X_1_	Male	0.304
X_4_	Hypertension	0.658
X_6_	Coronary heart disease	-0.061
X_7_	Diabetes	0.167
X_8_	Pulmonary disease	0.357

**Table 4:** Multivariate logistic regression analysis of risk factors associated with AIS after hip fracture

Model variables	B	S.E.	Wald	OR	95%CI	*p* Value
Male	0.623	0.272	5.264	1.865	1.095-3.177	**0.022**
Hypertension	1.039	0.304	11.669	2.827	1.557-5.131	**0.001**
Coronary heart disease	-0.493	0.315	2.442	0.611	0.329-1.134	0.118
Pulmonary disease	0.942	0.554	2.893	2.564	0.866-7.591	0.089
Diabetes	0.508	0.288	3.102	1.662	0.944-2.926	0.078

### Other complications of AIS after Hip Fracture

**Fig. 4:**
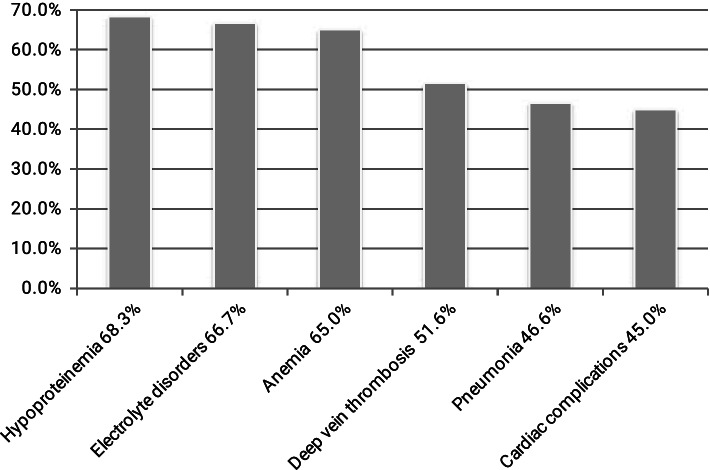
The proportion of other complications

In addition, we also observed the occurrence of other complications in AIS patients after hip fracture. Among 60 patients, most of patients were presenting with at least one complication, which consisted of hypoproteinemia (68.3%), electrolyte disorders (66.7%), anemia (65.0%), deep vein thrombosis (51.6%), pneumonia (46.6%), cardiac complications (45.0%).The specific information was presented in Fig. 4.

## Discussion

A total of 60 (3.8%) older hip fracture patients occurred AIS in all 1577 cases. Among 60 AIS patients, the number of female was higher than that of male, but the proportion of AIS was higher in male than female. The most cerebral infarction type was PACI and the most infarction lesion was single cerebral infarction in 60 AIS patients. Most of them were located on the left side. 81.7% of AIS patients had mild (NIHSS<4) AIS. Older patients with AIS after hip fracture were more frequently complicated by hypertension, previous stroke, diabetes and were more likely to have hypoproteinemia, electrolyte imbalance, anemia, deep vein thrombosis, cardiac complications and pneumonia. Combined with hypertension and male sex were associated with an increased risk of AIS after hip fracture in the elderly.

There were significantly more female among AIS cases in our study, which is due to the reason that female tend to live longer and are more likely to develop stroke and fracture in older people [17, 18]. The AIS proportion was higher in male than female in older fracture people in our study. Studies have shown that male sex is a significant risk factor for stroke [19], and the incidence of stroke in male before the age of 85 is higher than in female [20]. The infarctions were solitary cerebral infarction and were located on the left side in our study. The result was consistent with previously published results of Hedna VS et al [21]. Left-sided strokes might be mentioned more often because they cause obvious symptoms like aphasia, while strokes on the right side can cause less obvious symptoms like hemineglector spatial disorientation [22].We think that this can also be applied to patients with AIS after fracture.

The proportion of AIS after hip fracture was 3.8% in our study, which was consistent with prior study [23]. In our study, it was found that AIS patients after hip fracture were more frequently comorbid with chronic medical illness diseases, including hypertension, prior stroke and diabetes. Hypertension and diabetes are significant risk factors for the occurrence of cerebrovascular accidents (CVA) after hip fracture [24]. It has been observed that the carotid arteries of patients with diabetes are thicker and harder than those of the general population [25]. In addition, it has been reported that the carotid stiffness index of hypertensive patients is significantly higher than that of the general population [26]. Hypertension and diabetes accelerate the progression of atherosclerosis. Atherosclerosis is an important pathological cause of ischemic stroke. 30.0% of ischemic stroke are caused by carotid atherosclerosis [27]. The pathogenesis of ischemic stroke is considered to be arterial embolism caused by rupture of carotid plaque, ulceration, and platelet activation.

A study has shown that hip fracture is associated with increased risk of stroke [28]. Physical inactivity, fear and pain are common in the elderly hip fracture patients. In this case, the pre-existing cerebrovascular risk may be exacerbated. This may be associated with traumatic stress response. Fracture can lead to the release of stress hormones such as glucocorticoids, glucagon, epinephrine, thyroxine and others, which is called the “stress response” [29]. Stress response induces platelet aggregation and promotes microcirculation dysfunction and thrombosis [29]. A large number of inflammatory mediators caused by stress are released after hip fracture, and inflammation in the acute phase can affect thrombosis. Inflammation is usually related to oxidative stress. The strong oxidative activity can also destroy normal cell structure and promote the progress of cardiovascular and cerebrovascular diseases [30].

Patients with both fractures and strokes have a worse prognosis than those with single injury [31], and this may be related to their stronger traumatic stress response. Our observations showed that AIS patients after hip fractures suffered other adverse events such as hypoproteinemia, anemia, electrolyte disturbances,pneumonia, lower limb venous thrombosis and cardiovascular complications. Complications also affected each other, resulting in worse prognosis.

Hypoalbuminemia, electrolyte imbalance, anemia, deep vein thrombosis and pneumonia were common complications after bone fracture in our study. Factors such as excessive blood loss and pain after the fracture induce stress response. Stress response puts the body in a state of enhanced catabolism and decreased anabolism, which may be related to the occurrence of hypoproteinemia and electrolyte disorder [32]. The traumatic stress response leads to the release of hormones such as catecholamines [33], which increase pulmonary artery systolic pressure and initiate pulmonary vasoconstriction and pulmonary interstitial edema. Moreover, an excessive stress response would impair organism immunity. These factors lead to the occurrence of pneumonia. In addition,after traumatic fracture, stress can activate the endogenous and exogenous coagulation system, resulting in hypercoagulability and coagulation dysfunction. This significantly increases the risk of deep vein thrombosis. Stroke related disorders such as cognitive dysfunction, impaired consciousness, neurogenic vomiting, neurogenic dysphagia, and motor dysfunction also contribute to the development of hypoalbuminemia, electrolyte imbalance [34], deep vein thrombosis and pneumonia. The studies have shown that the occurrence of complications increases the risk of in-hospital death [35, 36].

The proportion of cardiac complications was the lowest in our study, but it was the most serious. The persistent stress of older hip fracture patients can cause myocardial hypoxia and increase the imbalance between oxygen supply and myocardial demand. This is a common and complex pathophysiological mechanism that causes the occurrence of cardiac complications [37]. There are several common risk factors for stroke and ischemic heart disease. Underlying heart diseases, such as atrial fibrillation, valve defects, or congestive heart failure, increase the risk of stroke. Stroke interferes with autonomic control and can easily lead to cardiac complications in patients [38].Cardiac complications in patients with fracture or stroke can be fatal for the patients [39]. All the occurrence of these complications after hip fracture is associated with traumatic stress, and we need to strengthen the management of patients' breathing, circulation, blood, and thrombus to attenuate the stress response and improve the quality of patients' survival.

Furthermore, after we performed multivariate logistic regression on patients participated in this study, the data showed that comorbid with hypertension and male sex were significant risk factors for the AIS after hip fracture in the elderly. The mechanism of stroke caused by hypertension was discussed above. For this reason, we propose the following recommendations. Firstly, elderly patients comorbid with hypertension should prevent falls and fractures in their daily life. Secondly, Elderly patients comorbid with hypertension should pay attention to monitoring blood pressure, blood lipids, and preventing the occurrence and development of cerebrovascular disease. In addition, for these elderly patients who develop fracture, we should strengthen management to reduce the stress reaction and avoid the occurrence of stroke and other complications.

### Limitations

There are some limitations to our study that warrant discussion. First, it is a retrospective nature of the study and the intrinsic limitation of design seems to be inescapable. Second, there is the possibility of selection bias owing to small sample size. The conclusions drawn cannot completely and reliably represent the general clinical characteristics. Third, patients with AIS after fracture surgery were not included in our study. Further study is needed to compare the different characteristics of AIS after hip fracture and AIS after surgery.

## Conclusions

Elderly patients with AIS after hip fracture more often comorbid with hypertension, prior stoke and diabetes. Older patients who had hypertension are more likely to have AIS after hip fracture. AIS patients after hip fracture are prone to have multiple complications under traumatic stress, and we should enhance the management of these patients to reduce the stress response and avoid occurrence of complications.

## Data Availability

The data used to support the findings of this study are available from Zhiqian Wang upon request.

## Abbreviations

AIS: Acute ischemic stroke
LASSO: Least absolute shrinkage and selection operator
TACI: Total anterior circulation infarction
PACI: Partial anterior circulation infarction
POCI: Posterior circulation infarction
LACI: Lacunar infarction
NIHSS: National Institutes of Health stroke scale
AUC: Area under the curve
CVA: Cerebrovascular accidents
